# Evaluation of Biomass and Chitin Production of *Morchella* Mushrooms Grown on Starch-Based Substrates

**DOI:** 10.3390/foods8070239

**Published:** 2019-07-01

**Authors:** Aikaterini Papadaki, Panagiota Diamantopoulou, Seraphim Papanikolaou, Antonios Philippoussis

**Affiliations:** 1 Laboratory of Edible Fungi, Institute of Technology of Agricultural Products (ITAP), Hellenic Agricultural Organization - Demeter, 1 Sofokli Venizelou Street, 14123 -Lykovryssi, 14123 Attiki, Greece; 2 Department of Food Science & Technology, Agricultural University of Athens, Iera Odos 75, 11855 Athens, Greece

**Keywords:** *Morchella*, morel mushrooms, bioprocess development, solid state fermentation, food processing, glucosamine, polysaccharides, bioactive compounds

## Abstract

*Morchella* sp. is one of the most expensive mushrooms with a high nutritional profile. In this study, the polysaccharide content of *Morchella* species was investigated. Specifically, mycelium growth rate, biomass production, sclerotia formation, and glucosamine and total polysaccharides content of six *Morchella* species grown on a starch-based media were evaluated. Submerged fermentations in potato dextrose broth resulted in a glucosamine content of around 3.0%. In solid-state fermentations (SSF), using potato dextrose agar, a high linear growth rate (20.6 mm/day) was determined. Increased glucosamine and total polysaccharides content were observed after the formation of sclerotia. Biomass and glucosamine content were correlated, and the equations were used for the indirect estimation of biomass in SSF with agro-industrial starch-based materials. Wheat grains (WG), potato peels (PP), and a mixture of 1:1 of them (WG–PP) were evaluated as substrates. Results showed that the highest growth rate of 9.05 mm/day was determined on WG and the maximum biomass yield (407 mg/g) on WG–PP. The total polysaccharide content reached up to 18.4% of dried biomass in WG–PP. The results of the present study proved encouraging for the efficient bioconversion of potato and other starch-based agro-industrial waste streams to morel biomass and sclerotia eliciting nutritional and bioactive value.

## 1. Introduction

Mushrooms are widely known for their taste and flavor presenting many functional properties, primarily due to their unique chemical composition. They are consumed either fresh or processed. For instance, mushroom powder is used as a food additive to increase the content of dietary fibers in foods or as a partial flour substitute in bakery products [1]. In addition to fresh or dried mushrooms, fungal mycelium is also a rich source of bioactive compounds with many functional properties and has been suggested as an alternative mushroom product for human consumption [2]. Agro-industrial wastes and side streams have been converted into various bioactive compounds, including polysaccharides and enzymes, through mushroom cultivation [3,4,5]. Mushroom-derived polysaccharides, such as glucans and chitin, have attracted research interest mainly due to their antioxidant, anti-inflammatory, antitumor, and immune-stimulation activity [6]. 

Chitin is a structural polysaccharide of the fungal cell wall, composed of β(1,4)-linked units of N-acetyl-d-glucosamine [7]. The chitin content of fungal mycelium can reach up to 42% (w/w, dry mass) depending on the fungal species, fermentation mode (submerged or solid-state fermentation), and the substrate [7,8,9]. Chitin and its deacetylated derivate, chitosan, are valuable compounds finding many applications, namely in food and pharmaceutical industry, due to their antimicrobial and antioxidant properties [7]. They have been used as functional food components, edible films [7], immobilization support material for enzymes [10], delivery system for food ingredients, such as carotenoids [11], and for the treatment of human diseases [12]. Chitin production reached around 28,000 tons in 2015 [13], mainly produced from by-products of the seafood industry [7,14]. The chemical extraction of glucosamine from seafood by-products requires high energy consumption and the use of strong chemical reagents. However, the main disadvantages of using seafood by-products, as glucosamine sources, are the seasonal and geographical limitations, the potential allergic effects of the product, and the formation of products with inconsistent composition and physicochemical properties [8,15]. Thus, chitin production by fungal fermentations has been suggested as an alternative source, as glucosamine can be produced in controlled conditions by simpler extraction process and using less-aggressive chemicals [8,14].

Morels (*Morchella* spp.), belonging to the Helvellaceae family of ascomycetes, are among the most desirable edible wild mushrooms. Researchers have suggested that the differences in the appearance of morels are due to environmental influences and that the mycelial mass determines the appearance or phenotype of morels in terms of color (yellow or black), yet not the shape. They have attracted research interest due to their commercial value, medicinal properties, and unique taste and flavor [16]. The artificial commercial cultivation of the fruit bodies of morels on various agro-industrial substrates is a difficult process, which is linked to the formation of a heterokaryotic sclerotium [17]. Published studies on the considerable morphological variations in the physiology of *Morchella* sp. in different carbon [18] and nitrogen sources [19] are available, however reports dealing with the production of high-added-value compounds by *Morchella* sp. are scarce. The most recent studies have demonstrated that polysaccharides from *Morchella* sp. present antioxidant [20,21] and antitumor [22] properties. Additionally, sclerotia from mushrooms are considered as an exceptional source of bioactive components characterized for their functional and medicinal properties, such as antitumor and anti-inflammatory activities. Lau and Abdullah [23] pointed out that the biological activities of the sclerotia seemed to be comparable to those of the mycelia. However, their formation and chemical composition are strongly dependent on the substrate and the fermentation conditions [23].

Many researchers have studied those characteristics of *Morchella* mushrooms in submerged fermentations (SmF) [24,25,26], still little is known about their behavior in solid-state fermentations (SSF) [27]. The aim of the present study was to determine the glucosamine and polysaccharide contents in the mycelia of different *Morchella* strains grown on starch-based substrates. Initially, six different *Morchella* strains were cultivated on commercial starch-based substrates. Biomass production, sclerotia formation, and glucosamine and polysaccharide contents were determined in SmF and SSF. Equations relating biomass production and glucosamine content were established and subsequently applied to estimate the biomass production during SSF on agro-industrial starch-based substrates. To the best of our knowledge, this is the first study reporting the relation between the biomass production and the glucosamine content of *Morchella* strains.

## 2. Materials and Methods 

### 2.1. Raw Materials

Wheat grains (WG) and wheat bran were purchased from the local market (Athens, Greece). Potato peels (PP), HERMES variety, were kindly provided by the potato processing industry Tasty Foods S.A. (Athens, Greece).

### 2.2. Morchella Strains, Media, and Culture Conditions

Experiments were carried out using six *Morchella* strains, belonging to the group of yellow and black morels (Table 1). All strains were obtained from the fungal AMRL (Athens Mushroom Research Laboratory) culture collection of the Laboratory of Edible Fungi, Institute of Technology of Agricultural Products (LEF, ITAP). *Morchella* strains were grown on potato dextrose agar (PDA; Merck, Darmstadt, Germany) plates at 26 ± 0.5 °C for seven days. PDA plates were maintained at 4 ± 0.5 °C and used as fermentation inoculums.

### 2.3. Submerged Fermentations on Commercial Substrates

SmF fermentations were initially conducted for the evaluation of biomass production in commercial starch- and glucose-based media. Potato dextrose broth (PDB) media was prepared by enriching the extract from 300 g/L potatoes with glucose 20 g/L and CaCO_3_ 2 g/L. In addition, a glucose-based media (GPYB; Glucose Peptone Yeast Broth) was also prepared consisting of: Glucose, 30 g/L; peptone, 3.5 g/L; yeast extract, 2.5 g/L; CaCO_3_, 2 g/L; KH_2_PO_4_, 1 g/L; MgSO_4_·7H_2_O, 0.5 g/L; CaCl_2_·2H_2_O, 0.3 g/L; MnSO_4_·H_2_O, 0.04 g/L; ZnSO_4_·7H_2_O, 0.02 g/L; and FeCl_3_·6H_2_O, 0.08 g/L. Both media were used for the SmF cultures in static conditions. Erlenmeyer flasks of 100 mL capacity, containing 20 mL of each liquid medium, were autoclaved for 20 min at 121 ± 0.5 °C, and subsequently inoculated with two agar disks of 6 mm diameter. Inoculum disks were cut from a seven-day-old growing colony on a PDA Petri dish. Static cultures were incubated at 26 ± 0.5 °C for 21 days. Triplicates were made for every sampling to determine biomass production, mycelium glucosamine content, polysaccharide content, and sclerotia formation. The experimental data were fitted by ORIGIN software (OriginPro 8, Originlab Corporation, Northampton, MA, USA).

### 2.4. Solid-State Fermentations on Commercial Substrates

SSF using PDA plates were employed for the evaluation of biomass production, radius growth rate, and glucosamine content of *Morchella* strains. Also, the cellophane technique was applied for the estimation of the dry weight of the fungal biomass, since it prevents penetration of hyphae into solid medium and makes the separation of the fungus possible [28,29]. Specifically, 20 mL of PDA, prepared as previously described and solidified by the addition of 20 g/L agar, was poured into Petri dishes (90 mm diameter), covered by polyethylene terephthalate (PET) membrane disks. PET membranes were boiled twice for 15 min in deionized water to remove plasticizers [30] and sterilized at 121 ± 0.5 °C for 20 min. Petri dishes were inoculated at the center with a 6 mm diameter mycelium plug and incubated at 26 ± 0.5 °C. At least three replicates per treatment and sampling were used to study the growth of tested strains regarding radius growth rate and sclerotia formation, biomass production, and its glucosamine and polysaccharide content. The experimental data were fitted by ORIGIN software (Northampton, MA, USA).

### 2.5. Solid-State Fermentations on Agro-Industrial Substrates

Wheat grains (WG), potato peels (PP), and a mixture of them (WG:PP, 1:1) were used for the solid-state cultivation of selected *Morchella* strains. Substrate content was 95% of WG, PP, or WG–PP, supplemented with 5% of wheat bran. PP and WG were washed to remove any wasteful materials. WG were boiled for 20 min and left to cool down. After drainage they were mixed with the previously moistened wheat bran to obtain ~65%–70% moisture content, while the pH ranged from 6.5 to 6.9 after addition of 0.2% (w/w) CaCO_3_. Petri dishes (150 mm diameter) were filled with the substrates and autoclaved for 20 min at 121 ± 0.5 °C. Inoculation was carried out with a 9 mm diameter agar disk and incubated at 26 ± 0.5 °C for 30 days. Mycelial growth rate as well as sclerotia formation and maturation was recorded daily. The mycelium concentration in the substrate was indirectly estimated through the regression equations of glucosamine vs. biomass, defined previously in the PDA–PET experiment.

### 2.6. Analytical Methods

#### 2.6.1. Mycelium Growth Rate

The radius growth rate (Kr) of mycelium (expressed in mm/day), was determined by fitting the growth parameters using the equation [31,32]:

$$r=Kr\cdot t+r_{0},$$

where r and r_0_ are the colony radius at time t and t_0_, respectively, and Kr is the constant growth rate. Measurements of colony diameter on the surface of SSF were taken in two perpendicular directions every 12 or 24 h, until the colony completely covered the Petri dish.

#### 2.6.2. Determination of Biomass and Sugar Consumption in Submerged Fermentations

Samples were withdrawn from SmF at specific intervals for the determination of biomass production and sugars consumption. Fungal biomass was separated from the culture broth by filtration (Whatman No. 2, Buckinghamshire, UK), washed twice with deionized water, and dried at 60 ± 0.5 °C until constant weight. The clear broth was used for the determination of reduced sugars by the 3,5-dinitro-2-hydroxy-benzoic acid (DNS) method [33] and total sugars content was estimated by the phenol–sulfuric acid method according to Dubois et al. [34].

#### 2.6.3. Determination of Biomass in Solid-State Fermentations

The glucosamine present in the fungal cell wall was used to monitor fungal biomass in SSF. Initially, a glucosamine standard curve (glucosamine vs. absorbance) was obtained using various concentrations of N-acetyl-D-glucosamine (Sigma-Aldrich, St. Louis, MO, USA). Subsequently, regression equations of the glucosamine content of each *Morchella* strain were determined using dry biomass (biomass vs. glucosamine). The chitin content of dried biomass was hydrolyzed into N-acetylglucosamine according to the method described by Scotti et al. [35]. Specifically, around 2 g of dry sample was mixed with 5 mL of 72% H_2_SO_4_ (Merck, Germany) followed by agitation (130 rpm) (rotary shaker, MPM Instruments Srl., M301-OR, Italy) for 30 min at room temperature. Then, samples were diluted with 54 mL deionized water and treated at 121 ± 0.5 °C for 2 h. The hydrolyzate was neutralized (pH 7.0) with a 10 N NaOH solution (Merck, Darmstadt, Germany) and further treated for the quantification of glucosamine [4]. 

The colorimetric method of Ride and Drysdale [9] was carried out for the determination of glucosamine content. An aliquot of 3 mL of hydrolyzate was obtained and an equal volume of 5% (w/v) NaNO_2_ (Merck, Germany) and 5% (w/v) KHSO_4_ (Merck, Germany) were added. The mixture was agitated for 15 min and then centrifuged (1500 × *g*, 2 °C, 2 min) (Hettich Micro22R, Tuttlingen, Germany). Then, 3 mL of the supernatant was obtained followed by addition of 1 mL of 12.5% (w/v) NH_4_SO_3_NH_2_ (Merck, Germany) and agitation for 5 min. Subsequently, 1 mL of freshly prepared 0.5% (w/v) 3-methyl-2-benzothiazolone hydrazone hydrochloride (MBTH; Sigma-Aldrich, St. Louis, MO, USA) was added, followed by heating in a boiling water bath for 3 min. The mixtures were cooled down and 1 mL of freshly prepared 0.5% (w/v) FeCl_3_ (Alfa Aesar, Kandel, Germany) was added to each sample. After standing for 30 min, the solution was centrifuged and the absorbance was read at 650 nm (Jasco V-530 UV/VIS spectrophotometer, Jasco, Tokyo, Japan). The same protocol (hydrolysis and analysis) was followed using the unfermented medium as a blank. In SSF experiment results were expressed as mg fungal biomass per g of dry substrate.

#### 2.6.4. Determination of Total Polysaccharide Content

The content of polysaccharides was determined following a modification of the anthrone method [36,37,38] using sucrose as a standard. Specifically, anthrone reagent (0.2 g/L) was prepared in an aqueous solution of H_2_SO_4_ (70%, w/v), then the mixture was boiled for 15 min and rapidly cooled. The reagent was kept in a dark and cool (4 °C) place for 24 h. For the determination of total polysaccharides (TP), the dried biomass was extracted with 10 mL of H_2_SO_4_ 70% for 30 min using an agitation rate of 130 rpm. Subsequently, 5 mL anthrone reagent was added to 1 mL of the extracted sample. The mixture was first cooled in water, then swirled, heated in a water bath at 60 ± 0.5 °C for 8 min, and rapidly cooled. The absorbance of samples was measured at 630 nm (Jasco, V-530 UV/VIS, Tokyo, Japan) after 1 h.

## 3. Results and Discussion

### 3.1. Submerged Fermentations

#### 3.1.1. Biomass Production, Glucosamine, and Total Polysaccharide Content

The quantification of fungal biomass is unfeasible in SSF due to the penetration of the hyphae into the solid substrate [8,39]. Among the different methods for monitoring fungal biomass in SSF, the estimation of glucosamine content is considered a representative indicator [38]. Therefore, SmF fermentations were initially carried out in PDB and GPYB to determine the relationship between glucosamine content and dry biomass.

*Morchella* strains were cultivated for 21 days in PDB and GPYB. In all cases, *Morchella* strains were able to consume more than 90% of the initial sugar concentration, except for AMRL 82 in GPYB (75%). The highest biomass production and the respective glucosamine and polysaccharide contents are shown in Table 2. Biomass production ranged from 9.3 to 11.1 g/L for yellow morels and from 9.4 to 14.2 g/L for the black ones. The glucosamine content varied between 2.3%–3.7% (*w/w*) for both yellow and black morels. Although, in most cases, higher biomass concentrations were observed in the GPYB medium (~10–14 g/L), the highest biomass productivity was attained in PDB for almost all strains (~1.3–1.7 g/L/day). The yield of biomass (Yx/s) based on the utilized substrate was calculated in order to establish the relationship between microbial growth and substrate consumption (Table 2). Biomass yield ranged around 0.41–0.43 g/g in all cases, except for AMRL 36 and AMRL 82, which was above 0.5 g/g in GPYB medium. This probably means that the starch-based substrate promoted biomass productivity. Previous studies highlighted that starch has been characterized as a superior carbon source for *Morchella* strains [40,41]. Zhang et al. [42] reported the ability of *Morchella esculenta* to degrade starch and upgrade the nutritional value of cornmeal during SSF; it was attributed to the high α-amylase production (215 U/g on the 20th cultivation day). Xing et al. [43] reported high biomass production (12.6 g/L) by the black morel *Morchella conica* grown on a synthetic sucrose-based medium. Other studies have reported lower concentrations, ranging from 2.6 to 10 g/L for both black and yellow morels using various carbon sources [25,40,44,45,46,47]. 

In this study, glucosamine content varied among *Morchella* strains, from 2.3% to 3.7% of fungal dry matter. Obviously, glucosamine content was dependent on the fungal strain and the composition of the substrate, which has been previously reported by other studies [9,38,39,48]. The chitin content of *Morchella* sp. has been reported to be around 16% [49], but the relation of fungal biomass with glucosamine content on agro-industrial substrates has not been reported.

The polysaccharides present in mycelium and fruiting bodies are classified as glycoconjugates and can be quantified by the anthrone method [50]. Table 2 presents the results of the TP content of *Morchella* mycelia grown in PDB and GPYB. The TP content of mushrooms was influenced by the strains; yellow morels had ~10.4% and black ~11.2 % w/w. The morels AMRL 36 and 63 presented the highest TP content (10.1%–12.2%) among all strains. Morel mushrooms are well known for their rich nutritional composition and, specifically, their sugar profile comprises mainly mannose (up to 43% w/w on a dry basis) and mannitol (up to 11.5%, w/w), followed by glucose, trehalose, fructose, and arabitol [51]. Dried biomass from different culture days was obtained for each strain and their glucosamine content was estimated. The results were correlated and the linear regression equations of glucosamine versus biomass are shown in Table 3. Results demonstrate that biomass production and glucosamine content were found to be highly correlated (R^2^ > 0.97). The aim was to use the equations to convert glucosamine content into mycelia biomass in the following SSF experiments. This approach has been already successfully applied in SSF of the medicinal mushroom *Lentinula edodes* [4].

### 3.2. Solid-State Fermentations on Commercial Substrates

PDA was selected for the evaluation of growth rate, biomass production, glucosamine content, and sclerotia formation in SSF, due to the higher biomass productivity of *Morchella* strains in SmF (Table 2). Morel strains AMRL 14 and AMRL 82 strains were excluded from SSF due to the lower biomass productivity observed in SmF (Table 2).

#### 3.2.1. Mycelial Growth Rate

**Figure 1:**
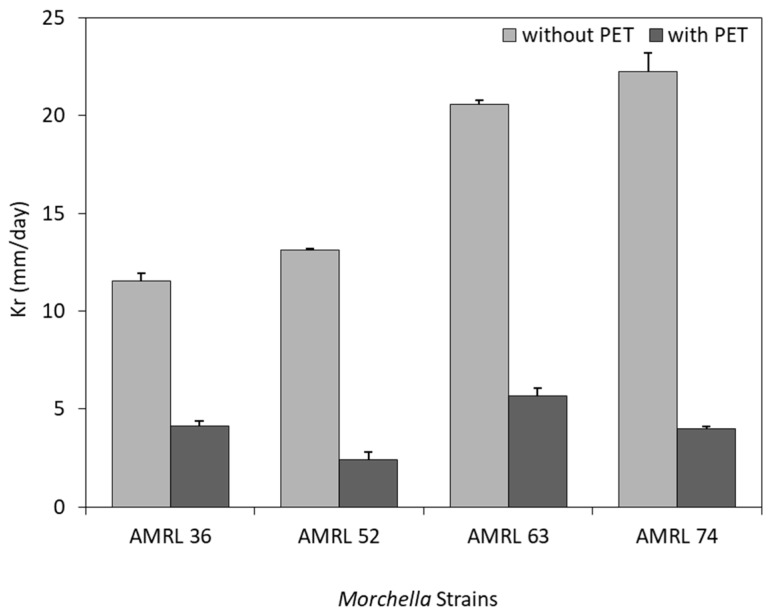
Growth rate of yellow (AMRL 36, 52) and black (AMRL 63, 74) morel strains during solid-state fermentations on potato dextrose agar (PDA), with and without polyethylene terephthalate (PET) membrane.

The mycelial growth rate of four *Morchella* strains was evaluated on PDA medium, with and without the addition of a PET membrane (Figure 1). In the case of SSF without PET membrane, strains from the black morels complex (AMRL 63 and 74) proved to grow faster than yellow morels. Among the black morels, the AMRL 74 strain presented the maximum growth rate (Kr) yielding 22.2 mm/day, whereas AMRL 52 presented the greater growth rate (13.1 mm/day) from the yellow morels complex. Brock [40] determined a growth rate of 21 mm/day for *M. esculenta* in SSF with glucose, as a carbon source. Winder [18] reported lower growth rates for the black morel strains compared to this study. More specifically, the growth rate was around 6 mm/day in SSF with sucrose and mannose as the substrate and 1 mm/day in PDA. Lower growth rates have been also determined for other mushrooms. More specifically, growth rates up to 6.6 mm/day for *Pleurotus* sp., 4.4 mm/day for *L. edodes*, and 18.8 mm/day for *Volvariella volvacea* have been reported during SSF on PDA [52]. In the case of SSF with PDA–PET, the growth rate was remarkably suppressed. The growth rate was reduced by 64%–82%, depending on the *Morchella* strain. The highest growth rate in PDA–PET was 5.7 mm/day from AMRL 63. *Morchella* strains have not been studied in SSF with the presence of the membrane. The only published study using PDA covered with a membrane reports a growth rate of 4.8 mm/day for *Morchella* [53]. Reeslev and Kjøller [54] reported that the presence of the membrane reduced the growth rate of the ascomycota *Paecilomyces farinosus* only by 8%. It could be assumed that the type of membrane can affect the growth rate. For instance, in this study a plastic membrane was employed, whereas Reeslev and Kjøller [54] used a cellulosic membrane.

#### 3.2.2. Sclerotia Formation

Sclerotia formation and maturation was studied on PDA substrate, with and without a PET membrane (Table 4). Maturation was expressed according to the size of sclerotia (immature ≤ 1 mm, mature > 1 mm), whereas the number of sclerotia was classified as follows: Few < 20, adequate > 20, and many > 50. In the case of PDA medium, morel strains AMRL 36, AMRL 52, and AMRL 74 produced few immature sclerotia at the eighth day of cultivation, whereas few mature sclerotia were formed only in the later fermentation stage (21st day). AMRL 63 exhibited more immature sclerotia compared with all the other strains. Immature and mature sclerotia of AMRL 63 appeared earlier.

Sclerotia formation was significantly impaired by the PET membrane. More matured sclerotia were formed in the case of AMRL 52 and AMRL 63 strains, as compared with PDA without PET. The number of immature and mature sclerotia was also promoted in all strains. Generally, starch has been found to promote sclerotia production [41]. The presence of PET induced a nutritional stress, which in turn promoted sclerotia formation [41]. The present study demonstrated that sclerotia formation was influenced by fermentation time, species, and culture conditions. Similar conclusions have also been mentioned by [41] for *Morchella* sp. Sclerotia are composed of large cells with thick walls and their presence is considered a precursor of fruiting body formation [41]. Additionally, their significance is based on their chemical composition as they are a rich source of bioactive compounds.

#### 3.2.3. Biomass Production, Glucosamine, and Total Polysaccharide Content

**Figure 2:**
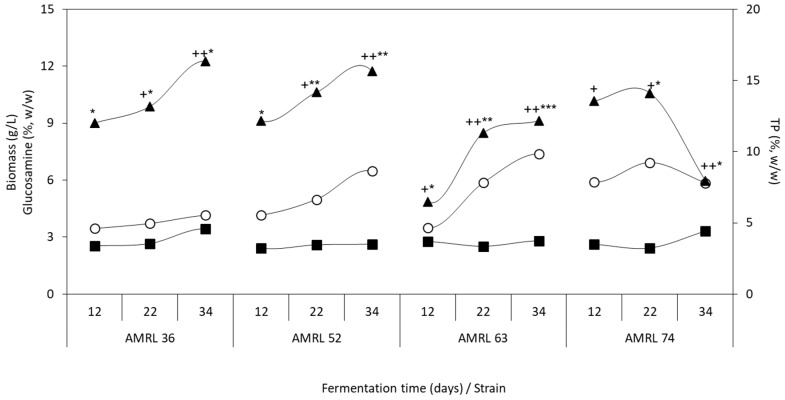
Biomass (○) and sclerotia production (+/*), glucosamine (■) and total polysaccharides (TP) (▲) content of yellow (AMRL 36, 52) and black (AMRL 63, 74) morel strains during solid-state fermentation (12th, 22nd, and 34th day) on potato dextrose agar (PDA), covered by polyethylene terephthalate (PET) membrane. The symbols +/* represents the number of immature/mature sclerotia, respectively (+/*; < 20, ++/**; > 20, +++/***; > 50).

The use of membranes as a separation method of mushroom biomass from solid substrates, allows the direct estimation of biomass, hence this method has been widely reported in literature [28]. In our study, PDA plates covered with PET were utilized and the biomass accumulated on the membrane surface was determined. Black morels achieved higher biomass concentrations than yellow morels on the PDA–PET substrate. In particular, the maximum biomass concentration was obtained for AMRL 63 (7.38 g/L) and AMRL 52 (6.47 g/L), both on the 34th day (Figure 2). These strains also presented the highest biomass concentration in PDB fermentation (Section 3.1.1, Table 2). Similar studies with *Morchella* strains have not been published, however the mycorrhizal fungi *Suillus collinitus* and *Pisolithus tinctorius* produced 7.1 and 5.4 g/L of biomass, respectively, in PDA with a membrane [29]. The glucosamine content was higher than 2.5% (w/w) for all *Morchella* strains (Figure 2). A positive correlation was found between biomass production and glucosamine content for all strains (R^2^ > 0.97 and R^2^ = 0.8 for AMRL 63). Similar glucosamine contents were obtained in SmF using PDB.

The age of the mycelium affected the TP content (Figure 2), which was found to be more than 15% for the yellow morel strains. The comparison with SmF in PDB revealed that TP content was enhanced in SSF. A positive correlation (R^2^ > 0.94) was found, between biomass production and TP content, for all strains (for AMRL 74 a positive correlation was found until the 22nd day). This is in agreement with previous results for other mushrooms [55]. On the top of that, Desgranges et al. [38] mentioned that TP content is increased as the age of the mycelium increases for the ascomycota *Beauveria bassiana*. Figure 2 showed that the TP content was influenced by sclerotia formation. In particular, increased TP content was observed as the number of mature and immature sclerotia increased. The determination of higher glucosamine and polysaccharide contents along with the appearance and maturation of sclerotia indicates that sclerotia are rich in chitin and polysaccharides. This has not been mentioned before for *Morchella* sp., however the presence of chitin and β-glucans was identified in sclerotia of *Pleurotus tuber-regium* [56]. The composition of the sclerotia of *Morchella* sp. should be further studied to identify the bioactive compounds and their biological activities.

The dried biomass was collected and correlated with glucosamine content. The linear regression equations were found to be the same, for each strain, as those indicated in Table 2 (PDB medium). These equations were further implemented for the determination of biomass formation in SSF with natural starch-based media.

### 3.3. Solid-State Fermentations on Agro-Industrial Substrates

SSF in WG, PP, and WG–PP were carried out using the morel strains AMRL 52 and AMRL 63, as they yielded higher biomass, TP content, and sclerotia formation in commercial substrates.

#### 3.3.1. Mycelial Growth Rate

**Figure 3:**
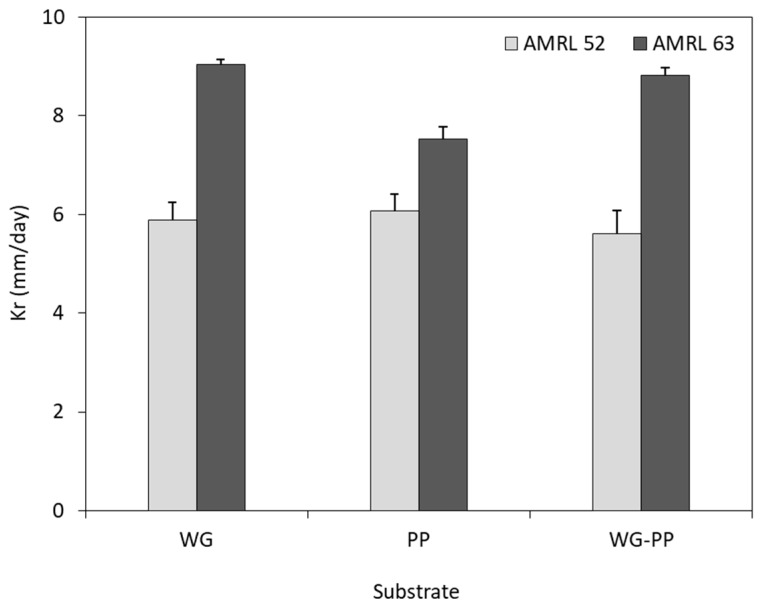
Growth rate of yellow (AMRL 52) and black (AMRL 63) morel strains during solid-state fermentation on wheat grains (WG), potato peels (PP), and a mixture of them (WG–PP, 1:1).

The growth rate of *Morchella* strains AMRL 63 and AMRL 52 was studied on WG, PP, and WG–PP substrates, as depicted in Figure 3. Both *Morchella* strains presented similar growth behavior in all substrates, with the black morel strain exhibiting faster growth rate than the yellow morel strain. Specifically, the highest growth rate of AMRL 63 was detected on WG substrate (9.0 mm/day) and WG–PP (8.8 mm/day), whereas AMRL 52 presented similar growth rates in all substrates (PP, 6.1 mm/day; WG, 5.9 mm/day; WG–PP, 5.6 mm/day).

The comparison of PDA and PP, WG, and WG–PP substrates demonstrated that AMRL 63 and AMRL 52 strains exhibited higher extension rates on agro-industrial substrates. This shows that starch-based substrates could be an alternative for the production of *Morchella* mycelium. 

The results clearly indicate that the substrate had a crucial role in the growth of *Morchella* strains. For instance, Alvarado-Castillo et al. [57] mentioned that growth rate was significantly affected by the type of grains used in SSF employed in jars. *Morchella* strains presented the highest mycelia growth (more than 30 cm^2^) in rye grains, followed by oats, wheat, and maize grains [57]. SSF of other mushrooms, such as *Pleurotus* sp., *L. edodes*, *Ganoderma* sp., and *V. volvacea* among others, have shown that the growth rate is highly dependent on the strain and the substrate [3,52]. *Pleurotus* sp., *Ganoderma* sp., and *Lentinula* sp. presented maximum growth rates ranging from 4.4 to 9.8 mm/day when cultivated on spent mushroom substrate, wheat straw, corn cobs, oak sawdust, and peanut shells) [3,52], whereas growth rate of *V. volvacea* reached 12.5 mm/day in wheat straw [52].

#### 3.3.2. Sclerotia Formation

Different outcomes were obtained in SSF of AMRL 52 and AMRL 63 strains in the WG, PP, and WG–PP substrates (Table 5), as compared to PDA substrate. The quantity of mature sclerotia was lower compared to the PDA–PET medium. *Morchella* strains formed sclerotia in WG and WG–PP substrate, whereas no sclerotia were observed in PP substrate. Among strains, AMRL 63 produced more mature and immature sclerotia on 30th day in WG–PP. It seems therefore that the mycelium growth rate is positively correlated to the sclerotia number. However, Alvarado-Castillo et al. [57] observed that growth rate was inversely related to sclerotia formation in SSF using various grains. Generally, the sclerotia formation of *Morchella* mushrooms is promoted in starch-based substrates [17], due to their content of rapidly metabolized sugars, such as starch and simple sugars. This has been also observed in SSF of *M. esculenta* using a co-substrate of wheat bran and corn starch [58].

#### 3.3.3. Biomass Production and Total Polysaccharide Content

**Figure 4:**
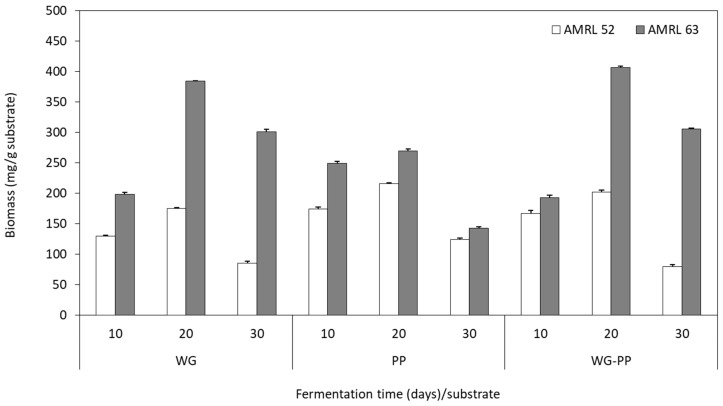
Estimated mycelium mass accumulation of yellow AMRL 52 and black AMRL 63 morel strains during solid-state fermentation on wheat grains (WG), potato peels (PP), and a mixture of them (WG–PP, 1:1).

An indirect estimation of biomass production was applied in SSF using starch-based agro-industrial substrates. In this case, the glucosamine content was determined and converted to biomass (mg per g of dried substrate) using the equations deriving from the SSF on PDA. As depicted in Figure 4, the highest biomass production of 407.1 and 384.6 mg/g were detected in WG–PP and WG substrates for the AMRL 63 strain. The morel strain AMRL 52 was favored by the PP substrate, presenting its maximum biomass (215.5 mg/g) on the 20th day. These results are in agreement with those obtained during SmF and SSF using PDB and PDA media, respectively, which confirmed that the black morel AMRL 63 is able to produce higher biomass concentrations than the yellow morel AMRL 52. Papinutti and Lechner [58] reported that *M. esculenta* produced 54 mg/g biomass in wheat bran. Similar studies dealing with the evaluation of biomass production using other mushrooms species have been previously reported. Specifically, *L. edodes* reached up to 510.3 mg/g during SSF of bean stalks [4]. Among *Ganoderma* strains, *G. resinaceum* showed a maximum biomass production of 151.23 mg/g during SSF in spent mushroom substrate. Moreover, *Pleurotus ostreatus* and *Pleurotus pulmonarius* presented biomass concentrations up to 141.62 mg/g when cultivated on the same substrate [3]. It is worth noting that there is a positive correlation between growth rate and biomass production, as observed also in SSF using commercial substrates. These results are not in accordance with previous findings reporting that growth rate and biomass production of *Ganoderma* and *Pleurotus* strains were negatively related [3,4,59]. 

The TP content of fermented agro-industrial substrates was similar for the black and yellow morels. Specifically, AMRL 52 and AMRL 63 mycelia achieved the highest TP content of 18.4% and 15.4% of dried biomass in PP and WG–PP, respectively. Previous studies have identified the bioactive compounds [60] deriving from *Morchella* mushrooms, showing that their functional properties are related to beneficial effects on human health [20,21,61]. The present results demonstrate the perspective for the production of bioactive compounds, such as glucosamine and polysaccharides, from *Morchella* sp. through the utilization of agro-industrial substrates.

## 4. Conclusions

The present study evaluated biomass production along with glucosamine and polysaccharides contents of black and yellow morel strains through the utilization of commercial and agro-industrial starch-based media. Glucosamine and polysaccharide mycelium contents were influenced by the age of the mycelia, presence of sclerotia, fermentation mode, type of substrate, and the strain. Linear regression equations between glucosamine and biomass of *Morchella* strains were reported for the first time. Biomass production and glucosamine content were found to be highly correlated, indicating that the determination of glucosamine content is a reliable indicator for the indirect estimation of *Morchella* biomass in SSF. In addition, high glucosamine and polysaccharide contents were correlated with high biomass production, presenting a R^2^ value higher than 0.9 for the majority of fungal strains. Conclusively, *Morchella* strains were able to produce biomass rich in glucosamine and polysaccharides in SSF using starchy materials. These results suggest that the mycelium and sclerotia of *Morchella* sp. could be used as an alternative source of bioactive compounds. SSF have present some difficulties regarding the recovery of bioactive compounds [8]. Alternatively, fermented substrates (e.g., cereals, fruit pomace) enriched with bioactive compounds can be directly utilized as food supplements. This has already been suggested for fermented solids rich in polyunsaturated fatty acids [62], thus it could be expanded for other bioactive compounds, including chitin and polysaccharides using mycelium from edible fungi. In addition, it has been indicated that the supplementation of culture media with various nutrients, including vegetable oils, improved biomass production and its glucosamine content [8]. Hence, optimization of fermentation conditions and exploitation of other culture media could contribute to higher glucosamine and polysaccharide contents in fungal mycelium.

## Figures and Tables

**Table 1:** *Morchella* strains used in the study.

Morels Group	*Morchella* Strain
Yellow morels	*M. rotunda* AMRL 14
	*M. vulgaris* AMRL 36
	*M. crassipes* AMRL 52
Black morels	*M. elata* AMRL 63
	*M. conica* AMRL 74
	*M. angusticeps* AMRL 82

AMRL: Athens Mushroom Research Laboratory.

**Table 2:** Maximum biomass production and glucosamine and total polysaccharide (TP) content of *Morchella* strains, during submerged fermentations in potato dextrose broth (PDB) and glucose-based broth (GPYB).

*Morchella* Strains	Medium	Time (days)	Biomass X (g/L)	Biomass Yield Yx/s (g/g)	Productivity P_X_ (g/L/day)	Glucosamine (%, w/w)	TP (%, w/w)
AMRL 14	PDB	14	10.7 ± 0.67	0.42	0.76	3.0 ± 0.07	9.6 ± 0.60
	GPYB	14	11.1 ± 0.80	0.41	0.79	3.7 ± 0.08	10.6 ± 0.03
AMRL 36	PDB	7	9.8 ± 0.45	0.41	1.40	2.5 ± 0.07	9.7 ± 0.02
	GPYB	7	10.2 ± 0.07	0.52	1.46	2.3 ± 0.08	10.8 ± 0.31
AMRL 52	PDB	7	9.3 ± 0.40	0.43	1.33	2.5 ± 0.09	10.1 ± 0.05
	GPYB	14	10.9 ± 0.35	0.43	0.78	3.1 ± 0.06	11.6 ± 0.30
AMRL 63	PDB	7	10.6 ± 0.45	0.43	1.51	2.3 ± 0.06	11.8 ± 0.12
	GPYB	14	14.2 ± 0.10	0.47	1.01	3.0 ± 0.04	12.2 ± 0.06
AMRL 74	PDB	7	11.8 ± 0.75	0.47	1.69	2.4 ± 0.04	10.3 ± 0.14
	GPYB	14	9.4 ± 0.97	0.32	0.67	3.1 ± 0.08	10.5 ± 0.10
AMRL 82	PDB	14	10.9 ± 0.55	0.42	0.78	3.5 ± 0.07	10.4 ± 0.30
	GPYB	14	12.4 ± 0.05	0.56	0.89	2.8 ± 0.05	10.9 ± 0.23

**Table 3:** Linear regression equations of glucosamine (mg) and mycelial biomass (g) of *Morchella* strains grown on potato dextrose broth (PDB) and glucose-based broth (GPYB).

*Morchella* Strains	Medium	Biomass (y)/Glucosamine (x)	Glucosamine (y)/Biomass (x)	R^2^
AMRL 14	PDB	y = 0.0335x − 0.0256	y = 29.499x + 0.8482	0.99
	GPYB	y = 0.0277x − 0.0046	y = 34.845x + 0.4457	0.97
AMRL 36	PDB	y = 0.0376x − 0.0064	y = 26.476x + 0.1888	0.99
	GPYB	y = 0.0336x − 0.0055	y = 29.308x + 0.2457	0.98
AMRL 52	PDB	y = 0.0369x − 0.0026	y = 26.151x + 0.2788	0.97
	GPYB	y = 0.0301x − 0.0002	y = 32.581x + 0.1026	0.98
AMRL 63	PDB	y = 0.0378x + 0.0020	y = 25.753x + 0.0513	0.97
	GPYB	y = 0.0328x − 0.0010	y = 30.057x + 0.0825	0.99
AMRL 74	PDB	y = 0.0385x + 0.0045	y = 25.627x - 0.0563	0.99
	GPYB	y = 0.0306x − 0.0124	y = 32.223x + 0.4669	0.99
AMRL 82	PDB	y = 0.027x − 0.0073	y = 36.685x + 0.3512	0.99
	GPYB	y = 0.0355x − 0.0219	y = 27.988x + 0.6530	0.99

**Table 4:** Sclerotia formation and maturation of yellow (AMRL 36, 52) and black (AMRL 63, 74) morel strains grown on PDA medium, with and without polyethylene terephthalate (PET) membrane.

Medium	Time (days)	Strains/Sclerotia Number and Maturation							
		AMRL 36		AMRL 52		AMRL 63		AMRL 74	
		I ^1^	M ^2^	I	M	I	M	I	M
PDA	8	+	−	+	−	+	−	+	−
	13	+	−	+	−	++	*	+	−
	21	+	*	+	*	++	*	+	*
PDA–PET	12	−	*	−	*	+	*	+	−
	22	+	*	+	**	++	**	+	*
	34	++	*	++	**	++	***	++	*

^1^ I: Immature sclerotia (≤ 1 mm); − no sclerotia, + few (< 20), ++ adequate (> 20), +++ many (> 50). ^2^ M: Mature sclerotia (>1 mm); − no sclerotia, * few (< 20), ** adequate (> 20), *** many (> 50).

**Table 5:** Sclerotia formation and maturation of yellow AMRL 52 and black AMRL 63 morel strains grown on wheat grains (WG), potato peels (PP), and a mixture of them (WG–PP, 1:1).

Medium	Time (days)	Strains/Sclerotia Number and Maturation			
		AMRL 52		AMRL 63	
		I ^1^	M ^2^	I	M
WG–PET	20	+	−	+	−
	30	++	−	+	*
PP–PET	20	−	−	−	−
	30	−	−	−	−
WG:PP–PET	20	+	*	++	*
	30	+	*	++	**

^1^ I: Immature sclerotia (≤ 1 mm); − no sclerotia, + few (< 20), ++ adequate (> 20), +++ many (> 50). ^2^ M: Mature sclerotia (>1 mm); − no sclerotia, * few (< 20), ** adequate (> 20), *** many (> 50).

## References

[1] Diamantopoulou P., Philippoussis A., Hui Y.H., Evranuz E.Ö., Bingöl G., Erten H., Jaramillo-Flores M.E. (2015). Cultivated Mushrooms: Preservation and Processing. Handbook of Vegetable Preservation and Processing.

[2] Carvajal A.E.S.S., Koehnlein E.A., Soares A.A., Eler G.J., Nakashima A.T.A., Bracht A., Peralta R.M. (2012). Bioactives of fruiting bodies and submerged culture mycelia of *Agaricus brasiliensis* (*A. blazei*) and their antioxidant properties. LWT-Food Sci. Technol..

[3] Economou C.N., Diamantopoulou P.A., Philippoussis A.N. (2017). Valorization of spent oyster mushroom substrate and laccase recovery through successive solid state cultivation of *Pleurotus*, *Ganoderma*, and *Lentinula* strains. Appl. Microbiol. Biotechnol..

[4] Philippoussis A., Diamantopoulou P., Papadopoulou K., Lakhtar H., Roussos S., Parissopoulos G., Papanikolaou S. (2011). Biomass, laccase and endoglucanase production by *Lentinula edodes* during solid state fermentation of reed grass, bean stalks and wheat straw residues. World J. Microbiol. Biotechnol..

[5] Philippoussis A., Zervakis G., Diamantopoulou P. (2001). Bioconversion of lignocellulosic wastes through the cultivation of the edible mushrooms *Agrocybe aegerita*, *Volvariella volvacea* and *Pleurotus* spp.. World J. Microbiol. Biotechnol..

[6] Choong Y.-K., Ellan K., Chen X.-D., Mohamad S.A. (2018). Extraction and Fractionation of Polysaccharides from a Selected Mushroom Species, *Ganoderma lucidum*: A Critical Review, Fractionation, Hassan Al- Haj Ibrahim. https://www.intechopen.com/books/fractionation/extraction-and-fractionation-of-polysaccharides-from-a-selected-mushroom-species-ganoderma-lucidum-a.

[7] Hamed I., Özogul F., Regenstein J.M. (2016). Industrial applications of crustacean by-products (chitin, chitosan, and chitooligosaccharides): A review. Trend Food Sci. Technol..

[8] Sitanggang A.B., Sophia L., Wu H.S. (2012). Aspects of glucosamine production using microorganisms. Int. Food Res. J..

[9] Ride J.P., Drysdale R.B. (1971). A chemical method for estimating *Fusarium oxysporum* f. *lycopersici* in infected tomato plants. Physiol. Plant Pathol..

[10] Zdarta J., Meyer A.S., Jesionowski T., Pinelo M. (2018). A General Overview of Support Materials for Enzyme Immobilization: Characteristics, Properties, Practical Utility. Catalysts.

[11] Tan C., Feng B., Zhang X., Xia W., Xia S. (2016). Biopolymer-coated liposomes by electrostatic adsorption of chitosan (chitosomes) as novel delivery systems for carotenoids. Food Hydrocoll.

[12] Cheung R.C.F., Ng T.B., Wong J.H., Chan W.Y. (2015). Chitosan: An Update on Potential Biomedical and Pharmaceutical Applications. Mar. Drugs.

[13] A Worldwide Market with a Strong Demand. http://sflyproteins.com/a-worldwide-market-with-a-strong-demand/.

[14] Wu T., Zivanovic S., Draughon F.A., Sams C.E. (2004). Chitin and Chitosan Value-Added Products from Mushroom Waste. J. Agric. Food Chem..

[15] Di Lena G., Annibate A.D., Sermanni G.G. (1994). Influence of the age and growth conditions on the mycelial chitin content of *Lentinus edodes*. J. Basic Microbiol..

[16] Prasad P., Chauhan K., Kandari L.S., Maikhuri R.K., Purohit A., Bhatt R.P., Rao K.S. (2002). *Morchella esculenta* (Guchhi): Need for scientific intervention for its cultivation in Central Himalaya. Curr. Sci..

[17] Volk T.J., Leonard T.J. (1990). Cytology of the life-cycle of *Morchella*. Mycol. Res..

[18] Winder R.S. (2006). Cultural studies of *Morchella elata*. Mycol. Res..

[19] Güller P., Arkan O. (2000). Cultural Characteristics of *Morchella esculenta* Mycelium on Some Nutrients. Turk. J. Biol..

[20] Xu N., Lu Y., Hou J., Liu C., Sun Y. (2018). A Polysaccharide Purified from *Morchella conica* Pers. Prevents Oxidative Stress Induced by H_2_O_2_ in Human Embryonic Kidney (HEK) 293T Cells. Int. J. Mol. Sci..

[21] Yang C., Zhou X., Meng Q., Wang Μ., Zhang Y., Fu S. (2019). Secondary Metabolites and Antiradical Activity of Liquid Fermentation of *Morchella* sp. Isolated from Southwest China. Molecules.

[22] Liu C., Sun Y., Mao Q., Guo X., Li P., Liu Y., Xu N. (2016). Characteristics and Antitumor Activity of *Morchella esculenta* Polysaccharide Extracted by Pulsed Electric Field. Int. J. Mol. Sci..

[23] Lau B.F., Abdullah N., Petre M. (2016). Sclerotium-Forming Mushrooms as an Emerging Source of Medicinals: Current perspectives. Mushroom Biotechnology, Developments and Applications.

[24] Gilbert F. (1960). The submerged culture of *Morchella*. Mycologia.

[25] Kaul T.N. (1978). Physiological studies on *Morchella* spp. I. Carbon utilization. Bull. Bot. Soc. Bengal..

[26] Kosaric N., Miyata N. (1981). Growth of morel mushroom mycelium in cheese whey. J. Dairy Res..

[27] Philippoussis A., Balis C., Elliott T.J. (1995). Studies on the morphogenesis of sclerotia and subterranean mycelial network of ascocarps in “*Morchella*” species. Science and Cultivation of Edible Fungi, Proceedings of the 14th international congress on the science and cultivation of edible fungi, Oxford, England.

[28] Ang T.N., Ngoh G.C., Chua A.S.M. (2013). Development of a novel inoculum preparation method for solid-state fermentation-Cellophane film culture (CFC) technique. Ind. Crop. Prod..

[29] Araujo A.A., Roussos S. (2002). A technique for mycelial development of ectomycorrhizal fungi on agar media. Appl. Biochem. Biotechnol..

[30] Robson G.D., Bell S.D., Kuhn P.J., Trinci A.P.J. (1987). Glucose and penicillin concentrations in agar medium below fungal colonies. J. Gen. Microbiol..

[31] Dantigny P., Guilmart A., Bensoussan M. (2005). Basis of predictive mycology. Int. J. Food Microbiol..

[32] Baranyi J., Roberts T.A., McClure P. (1993). A non-autonomous differential equation to model bacterial growth. Food Microbiol..

[33] Miller G.L. (1959). Use of dinitrosalicylic acid reagent for determination of reducing sugars. Anal. Chem..

[34] Dubois M., Gilles K.A., Hamilton J.K., Rebers P.A., Smith F. (1956). Colorimetric method for determination of sugars and related substances. Anal. Chem..

[35] Scotti C.T., Vergoignan C., Feron G., Durand A. (2001). Glucosamine measurement as indirect method for biomass estimation of *Cunninghamella elegans* grown in solid state cultivation conditions. Biochem. Eng. J..

[36] Bailey R.W. (1958). The reaction of pentoses with anthrone. Biochem. J..

[37] De Bruyn J.W., Van Keulen H.A., Ferguson J.H.A. (1968). Rapid method for the simultaneous determination of glucose and fructose using anthrone reagent. J. Sci. Food Agric..

[38] Desgranges C., Vergoignan C., Georges M., Durand A. (1991). Biomass estimation in solid state fermentation I. Manual biochemical methods. Appl. Microbiol. Biotechnol..

[39] Roche N., Venague A., Desgranges C., Durand A. (1993). Use of chitin measurement to estimate fungal biomass in solid state fermentation. Biotechnol. Adv..

[40] Brock D.T. (1951). Studies on the Nutrition of *Morchella esculenta Fries*. Mycologia.

[41] Stott K., Mohammed C. (2004). Specialty Mushroom Production Systems: Maitake and Morels. A report for the Rural Industries Research and Development Corporation.

[42] Zhang G.P., Zhang F., Ru W.M., Han J.-R. (2010). Solid-state fermentation of cornmeal with the ascomycete *Morchella esculenta* for degrading starch and upgrading nutritional value. World J. Microbiol. Biotechnol..

[43] Xing Z., Sun F., Liu J. (2004). Studies on the submerged-cultured conditions of *Morchella conica*. Acta Edulis Fungi.

[44] Buswell A.J., Chang S. (1994). Biomass and extracellular hydrolytic enzyme production by six mushroom species grown on soybean waste. Biotechnol. Lett..

[45] Bensoussan M., Tisserand E., Kabbaji W., Roussos S. (1995). Partial characterization of aroma produced by submerged culture of morel mushroom mycelium. Cryptog. Mycol..

[46] Meng F., Liu X., Jia L., Song Z., Deng P., Fan K. (2010). Optimization for the production of exopolysaccharides from *Morchella esculenta* SO-02 in submerged culture and its antioxidant activities in vitro. Carbohydr. Polym..

[47] Xu H., Sun L.-P., Shi Y.-Z., Wu Y.-H., Zhang B., Zhao D.-Q. (2008). Optimization of cultivation conditions for extracellular polysaccharide and mycelium biomass by *Morchella esculenta* As51620. Biochem. Eng. J..

[48] Sparringa A.R., Owens D.J. (1999). Glucosamine content of tempe mould, *Rhizopus oligosporus*. Int. J. Food Microbiol..

[49] Ruíz-Herrera J., Osorio E. (1974). Isolation and chemical analysis of the cell wall of *Morchella* sp.. Antonie van Leeuwenhoek.

[50] Broecker F., Seeberger P.H. (2017). Identification and Design of Synthetic B Cell Epitopes for Carbohydrate-Based Vaccines. Methods Enzymol..

[51] Tietel Z., Masaphy S. (2018). True morels (*Morchella*)—Nutritional and phytochemical composition, health benefits and flavor: A review. Crit. Rev. Food Sci. Nutr..

[52] Zervakis G., Philippoussis A., Ioannidou S., Diamantopoulou P. (2001). Mycelium growth kinetics and optimal temperature conditions for the cultivation of edible mushroom species on lignocellulosic substrates. Folia Microbiol..

[53] Masaphy S. (2004). Effect of CaCO_3_ on *Morchella* growth and sclerotia formation. Int. Soc. Mushroom Sci..

[54] Reeslev M., Kjøller A. (1995). Comparison of biomass dry weights and radial growth rates of fungal colonies on media solidified with different gelling compounds. Appl. Environ. Microbiol..

[55] Petre V., Petre M., Rusea I., Stǎnicǎ F., Petre M. (2016). Biotechnological recycling of fruit tree wastes by solid-state cultivation of mushrooms. Mushroom Biotechnology, Developments and Applications.

[56] Cheung P.C.K., Lee M.Y. (2000). Fractionation and Characterization of Mushroom Dietary Fiber (Nonstarch Polysaccharides) as Potential Nutraceuticals from Sclerotia of *Pleurotus tuber-regium* (Fries) Singer. J. Agric. Food Chem..

[57] Alvarado-Castillo G., Mata G., Pérez-Vázquez A., Martínez-Carrera D., Tablada M.E.N., Gellardo-López F., Osorio-Acosta F. (2011). *Morchella* sclerotia production through grain supplementation. Interciencia.

[58] Papinutti L., Lechner B. (2008). Influence of the carbon source on the growth and lignocellulolytic enzyme production by *Morchella esculenta* strains. J. Ind. Microbiol. Biotechnol..

[59] Philippoussis A., Diamantopoulou P., Petre M., Berovic M. (2012). Exploitation of the biotechnological potential of agro-industrial by-products through mushroom cultivation. Mushroom Biotechnology and Bioengineering.

[60] Lo Y.C., Lin S.Y., Ulziijargal E., Chen S.Y., Chien R.C., Tzou Y.J., Mau J.L. (2012). Comparative study of contents of several bioactive components in fruiting bodies and mycelia of culinary-medicinal mushrooms. Int. J. Med. Mushrooms.

[61] Pinto M.R., Barreto-Bergter E., Taborda C.P. (2008). Glycoconjugates and polysaccharides of fungal cell wall and activation of immune system. Braz. J. Microbiol..

[62] Ochsenreither K., Glück C., Stressler T., Fischer L., Syldatk C. (2016). Production Strategies and Applications of Microbial Single Cell Oils. Front. Microbiol..

